# Massive mass embolism detected by transesophageal echocardiography in bone cement implantation syndrome: a case report

**DOI:** 10.1186/s40981-019-0225-2

**Published:** 2019-01-24

**Authors:** Yuki Izumi, Satoshi Ishihara, Ivor Cammack, Ikuko Miyawaki

**Affiliations:** 1 Department of Anesthesiology and Critical Care, Teine Keijinkai Hospital, 006-0085 12-1-40, 1-jo, Maeda, Teine-ku, Sapporo, Hokkaido Japan; 2 Department of Clinical Residency, Teine Keijinkai Hospital, 006-0085, 12-1-40, 1-jo, Maeda, Teine-ku, Sapporo, Hokkaido Japan; 3 Department of Anesthesiology and Critical Care, Kobe City Medical Center General Hospital, 650-0047, 2-1-1, Minatojimaminamimachi, Chuo-ku, Kobe, Hyogo Japan

**Keywords:** Bone cement implantation syndrome (BCIS), Cemented orthopedic surgeries, Pulmonary embolism (PE), Transesophageal echocardiography (TEE)

## Abstract

Bone cement implantation syndrome (BCIS) is a known complication in patients undergoing cemented orthopedic surgeries; however, the etiology and pathophysiology of BCIS are not fully understood. We report the case of a patient who developed pulseless electrical activity (PEA) due to BCIS after cemented femoral head replacement. Transesophageal echocardiography (TEE) during PEA revealed a massive embolus extending from the main pulmonary artery to the inferior vena cava. Of note, this embolus disappeared completely and rapidly after return of spontaneous circulation. TEE proved to be useful in the diagnosis and management of this case of PEA.

**Electronic supplementary material:**

The online version of this article (10.1186/s40981-019-0225-2) contains supplementary material, which is available to authorized users.

## Background

Bone cement implantation syndrome (BCIS) is characterized by hypoxia, hypotension, cardiac arrhythmias, increased pulmonary vascular resistance, and cardiac arrest. BCIS is fatal in 0.6–1% of cases [1]. It is a known complication in patients undergoing cemented orthopedic surgeries; however, the etiology and pathophysiology of BCIS are not fully understood. We report the case of BCIS diagnosed by transesophageal echocardiography (TEE) during pulseless electrical activity (PEA).

## Case presentation

A 93-year-old woman, with no history of cardiac disease or venous thrombosis, presented with left neck femur fracture. She was scheduled for cemented femoral head hemiarthroplasty. Pre-operative evaluation showed that she was generally well, with no signs of deep vein thrombosis (DVT) such as tenderness or swelling of the legs. The D-dimer was slightly raised at 5.43 μg/ml, but ultrasound examination of the lower limbs was not performed. After arrival at the operating room, she was monitored with electrocardiography, non-invasive blood pressure, and pulse oximetry. An arterial line was inserted in the radial artery. General anesthesia was induced with propofol, fentanyl, and rocuronium and maintained with desflurane and remifentanil, and she was intubated without complication. She was placed in the right lateral decubitus position. She remained stable until the prosthesis was inserted and cemented when she suddenly developed hypotension. PEA was diagnosed by the absence of carotid pulse and sinus rhythm on the electrocardiogram. Cardiopulmonary resuscitation (CPR) was initiated, and three doses of 1 mg epinephrine were administered. Spontaneous circulation returned after 8 min. A TEE probe was inserted during CPR to evaluate the cause of PEA. TEE detected a huge mobile mass filling the main pulmonary artery and its branches causing obstruction from the inferior vena cava to the right atrium. A video recording of this mass can be seen on Video 1. Sixty minutes later, the mass had completely disappeared (Video 1). Postoperatively, she remained sedated and intubated and was transferred to the intensive care unit (ICU). Continuous intravenous infusion of 0.1 μg/kg/min norepinephrine was required for 12 h to correct persistent hypotension. She was extubated 36 h after surgery and remained 4 days in the ICU. She was discharged 33 days post-operatively, with no apparent neurologic sequelae. Her hospital stay was prolonged due to development of a small lower limb deep vein thrombosis, but it was not thought that this was related to her large intra-operative embolism.

## Discussion

We learned that intravascular emboli caused by BCIS can extend from the main pulmonary artery to the inferior vena cava and can disappear completely within approximately 60 min. Immediate TEE is a useful adjunct for the diagnosis and management of cardiopulmonary collapse or cardiac arrest in patients undergoing non-cardiac surgery.

Previous reports have already documented embolic masses due to BCIS, seen with TEE. However, these are usually localized in the inferior vena cava, the right atrium, or right ventricle [2–5]. In these reports, embolic masses have not been followed by TEE re-examination. Embolic masses have been captured by TEE in some prospective clinical studies of BCIS; however, embolic masses captured by TEE during cardiopulmonary collapse or cardiac arrest in BCIS are rarely reported [3]. The etiology and pathophysiology of BCIS is not fully understood but recent research has investigated the mechanism of embolization [2]. Introduction of bone cement into the medullary space during prosthesis insertion leads to increase in intra-medullary pressure, resulting in embolization of fat, marrow, cement particles, air, bone particles, and aggregates of platelets and fibrin [1, 2]. Several mechanisms such as histamine release and endogenous cannabinoid vasodilation have also reported [2]. In addition, emboli can release vasoactive or pro-inflammatory substances that increase pulmonary vascular resistance and other mediators that decrease systemic vascular resistance [2]. In the present case, BCIS was caused by embolus; however, other mechanisms could also have co-existed.

Few previous reports have demonstrated the spontaneous disappearance of emboli in BCIS. Unlike chronic emboli, which may be very dense and difficult to breakdown, the acute emboli in BCIS are formed very quickly and, as a result, may also breakdown easily. Also, chronic emboli are usually made of a homogenous material, such as a platelet aggregate, but in the case of BCIS, there may be a mixed component embolus containing fat, marrow, cement particles, air, and bone particles. We hypothesize that such heterogenous emboli may also breakdown more rapidly.

Immediate TEE is useful for the diagnosis and the management of cardiopulmonary collapse or cardiac arrest in patients undergoing non-cardiac surgery [6–8]. It is preferable over transthoracic echocardiography because it can be performed without interrupting chest compression and better views can be obtained. The causes of cardiac arrest, including acute myocardial infarction, cardiac tamponade, pulmonary embolism, aortic dissection, papillary muscle rupture, ruptured aorta, and cardiac arrhythmia, can be correctly diagnosed with TEE in 90% of patients [6]. In the present case, we considered establishing extracorporeal membrane oxygenation (ECMO) during CPR because of the diagnosis of pulmonary embolism (PE) by TEE. However, TEE re-examination post-return of spontaneous circulation observed that the mass had disappeared completely. Thus, we were able to avoid ECMO.

PE was also considered likely in this case. Patients with femoral neck fracture are at increased risk of venous thromboembolism (VTE). In patients following hip fracture without thromboprophylaxis, the incidences of VTE, proximal thromboembolism, and fatal PE range from 42 to 50%, 20–27%, and 0.6–7.5% respectively [9]. In the present case, however, it is unlikely that such a large thrombus could have embolised from the calf, and then disappear so quickly after the return of spontaneous circulation. Previous reports show that most cardiopulmonary collapse caused by BCIS occurs within approximately 3 min of cementation [10]. This suggests strongly that the pulmonary embolism of the present case was due to BCIS.

## Conclusion

The present case demonstrates that with BCIS, massive emboli can extend from the main pulmonary artery to the inferior vena cava and can disappear completely in a short time. Cardiopulmonary collapse or cardiac arrest shortly after cementation is suggestive of BCIS. In this situation, TEE can help with diagnosis and influence management decisions. Further research is required to investigate the etiology and pathophysiology of BCIS.

## Additional file

Additional file 1: **Video S1.** The midesophageal ascending aortic short-axis view shows a huge mobile mass embolism obstructing main pulmonary artery and its branches. The midesophageal four chamber view and modified transgastric hepatic vein view show a mobile mass embolism filling of right atrium and inferior vena cava. Sixty minutes later, the mass embolism has completely disappeared. (MP4 1290 kb)

## Abbreviations

BCIS: Bone cement implantation syndrome
CPR: Cardiopulmonary resuscitation
DVT: Deep vein thrombosis
ECMO: Extracorporeal membrane oxygenation
ICU: Intensive care unit
PE: Pulmonary embolism
PEA: Pulseless electrical activity
TEE: Transesophageal echocardiography
VTE: Venous thromboembolism
